# Learning New Movements Depends on the Statistics of Your Prior Actions

**DOI:** 10.1371/journal.pbio.0040354

**Published:** 2006-09-12

**Authors:** Mason Inman

It’s tough to learn to drive on the other side of the road than you’re used to—just ask any American driving in London for the first time. Yet there’s little you can do consciously to change such habits; you simply have to take the time to practice with the new set of rules. Now a new study shows that if people are given appropriate cues and learn tasks in a certain order, they can learn new rules more quickly and call them up at the right time.

John Krakauer and colleagues wanted to figure out how people learn new movements, like controlling a computer mouse, and then commit them to motor memory, to be reactivated when necessary. But learning can be a double-edged sword: sometimes learning one task makes it easier to learn another; other times the skills you’ve already picked up make it harder to learn something new.

It’s been hard for researchers to understand when a person learning a new task will benefit from their past experience, and when history is a hindrance. It’s also not clear what cues people might use to call up the appropriate set of rules from what they’ve learned before. This study shows that the benefit or hindrance afforded by training is itself dependent on the history of the learner, and that this history-dependent pattern obeys Bayesian statistics—which use prior knowledge to predict an outcome. Importantly, the statistics that matter seem to be the history of motion of various body parts.

Krakauer and colleagues thought that perhaps people are unconsciously influenced by the history of how they have used their various body parts to learn a movement, and that this memory strongly influences how they will learn other new tasks. In effect, no learner is a blank sheet, but approaches every new task with strong biases. These biases are the accumulated history of how they have used their body in the past.

To test this possibility, they had people control a computer cursor using either just wrist movements or their whole arm and shoulder, with the wrist immobilized. Participants had to learn to adjust to having their control over the cursor manipulated: if they tried to move the cursor up, for example, it would move up and to the right; moving the cursor down would send it down and to the left. Krakauer and colleagues found that when a person learned to cope with one rotation first (with the arm), it helped them learn to cope more quickly with the same rotation with their wrist. But the reverse was not true: learning first with the hand did not aid learning with the arm. So, when learning new movements, the body faces the problem of deciding which body part to give credit for learning a task, the researchers argue. Since movements of the arm also include moving the wrist and hand with it, then learning with the arm usually affects learning with the wrist and hand, too. But during most wrist movements, the arm is relatively still, so learning at the wrist stays at the wrist.

Then, in more-complex experiments, the researchers showed they could block generalization from the arm to the wrist. In these experiments, they had people learn one rotation—say, a clockwise one—using their wrist, and then the opposite rotation (counter-clockwise) using their arm. This learning of a clockwise rotation with the wrist, then counter-clockwise with the arm, did not disrupt what had been already learned at the wrist because testing again with clockwise rotation with the wrist showed that people could call up their previous training—interference had been blocked. So people were able to acquire two opposite rules, as long as they learned them with different body parts.

**Figure:**
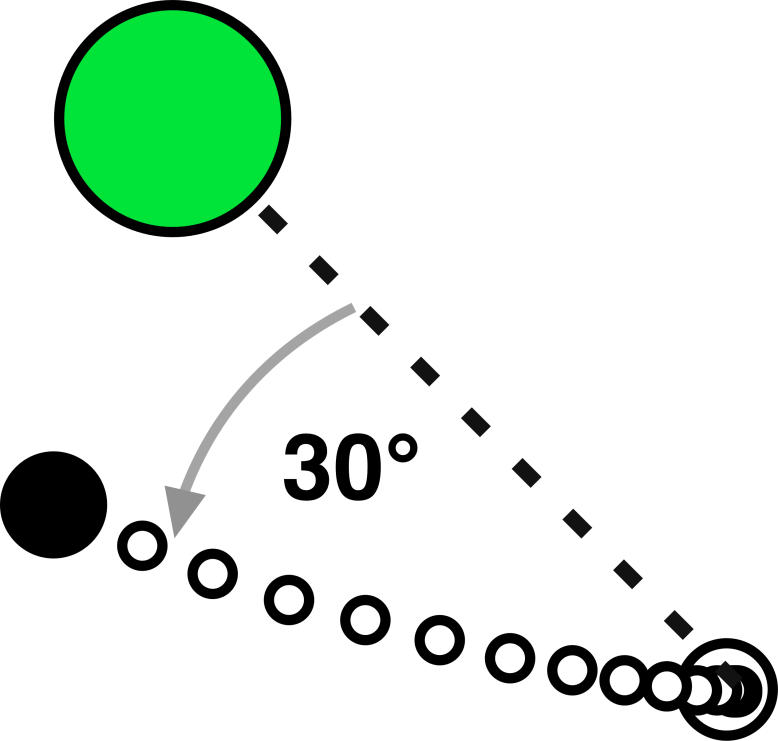
To study the effects of prior training on motor learning, the authors trained subjects to reach to an endpoint at a 30° angle from a visual target with either a wrist or an arm movement.

In a similar test, people went through the same first two steps: clockwise rotation at the wrist, then counter-clockwise rotation with the arm. Then they tried to learn counter-clockwise rotation with the wrist—but they were no better than novices at this–transfer had been blocked. So both experiments showed that previous training at the wrist blocked the generalization of learning from arm to wrist that would otherwise have occurred. It seems that using different body parts to learn different rules is enough for people to keep the rules separate.

Krakauer and colleagues bolstered their experimental findings by comparing them with a statistical model of movements of the arm and wrist. The model uses a Bayesian approach, where previous experience and new data are combined to form a new parameter estimate. A key aspect of this approach is that greater uncertainty about the parameter leads to faster learning. Crucially, in their model, the investigators assumed that the majority of movements with the arm also include moving the wrist, but not vice versa. This led to a situation where the uncertainty in the parameter estimate—the imposed rotation—depended not only on the current limb context but also on the history of training in previous limb contexts. The model was able to reproduce most of the effects they saw in the experiments, such as the finding that learning would transfer from arm to wrist, but not vice versa, and blocking of generalization.

Together, these studies suggest that when learning two different rotations, learning the second rotation does not disrupt consolidation of the memory of the first, Krakauer and colleagues argue. Instead, the history of training with each body part and how the body parts work together determine whether a person can learn two different mappings and call them up at the appropriate times.

